# Kunihiro Hattori, Tomoyuki Igawa, and Takehisa Kitazawa share Lasker Award honors for revolutionary hemophilia A therapy

**DOI:** 10.1172/JCI212215

**Published:** 2026-09-09

**Authors:** Robert Flaumenhaft

**Affiliations:** Division of Hemostasis and Thrombosis, Department of Medicine, Beth Israel Deaconess Medical Center, Harvard Medical School, Boston, Massachusetts, USA.

The 2026 Lasker~DeBakey Clinical Medical Research Award has been awarded to Kunihiro Hattori, Tomoyuki Igawa, and Takehisa Kitazawa for the development of a bispecific antibody that serves as a surrogate for factor VIIIa, bridging factors IXa and X to restore hemostasis in hemophilia A. This humanized bispecific monoclonal antibody, named emicizumab, has revolutionized hemophilia management because of its long half-life, subcutaneous bioavailability, and low immunogenicity. Furthermore, emicizumab can be used in the presence FVIII inhibitors, which complicate approximately 30% of cases of hemophilia A treated with recombinant FVIII.

## The clinical context

Hemophilia A is an X-linked deficiency in factor VIII (FVIII) that affects approximately 1 in 5,000 male births. Severe hemophilia A is associated with intracranial bleeds in infants, life-threatening bleeding with minor injuries, hemarthrosis leading to joint deformity, spontaneous soft-tissue and muscle hematomas, and internal hemorrhage. Recurrent, unchecked hemarthroses drive a self-perpetuating cycle of synovitis and rebleeding that culminates in hemophilic arthropathy in roughly 90% of severely affected patients by their second or third decade of life if untreated. Before the introduction of replacement therapy, patients with severe hemophilia A had a life expectancy of only approximately 11–13 years.

## FVIIIa replacement therapy: triumph and tragedy

The history of replacement therapy in hemophilia A is one characterized by both remarkable advances and devastating complications. In 1937, Patek and Taylor published in the *Journal of Clinical Investigation* that intravenous injection of a globulin fraction from normal plasma was capable of shortening the prolonged clotting time observed in three patients with hemophilia (1). The process for producing cryoprecipitate, which is highly enriched in FVIII, and its use in hemophilia was described in 1965 (2). Freeze-dried FVIII concentrates were subsequently used in the late 1960s and 1970s and evolved into highly purified FVIII products later in the 1970s and 1980s, enabling the advent of prophylaxis programs. Tragically, exposure to pooled, plasma-derived concentrates that were not virally inactivated in the late 1970s and early 1980s resulted in HIV infections in 60%–70% of and hepatitis C virus infections in nearly all people with severe hemophilia A (3). In 1984, FVIII was cloned (4–7), and 5 years later, the first use of recombinant FVIII in 2 patients with hemophilia A was reported (8). Recombinant FVIII products largely eliminated the risk of transmitting human blood-borne viruses. However, replacement therapy remains complicated by the development of neutralizing anti-FVIII antibodies, termed FVIII inhibitors. Inhibitors develop in approximately 30% of previously untreated patients with severe hemophilia A after FVIII exposure. In addition, the 8- to 12-hour half-life of standard-half-life recombinant FVIII products necessitates frequent intravenous infusions for prophylaxis. In young children, placement of a central venous access device is often required.

## An inspirational step

**Figure 1:**
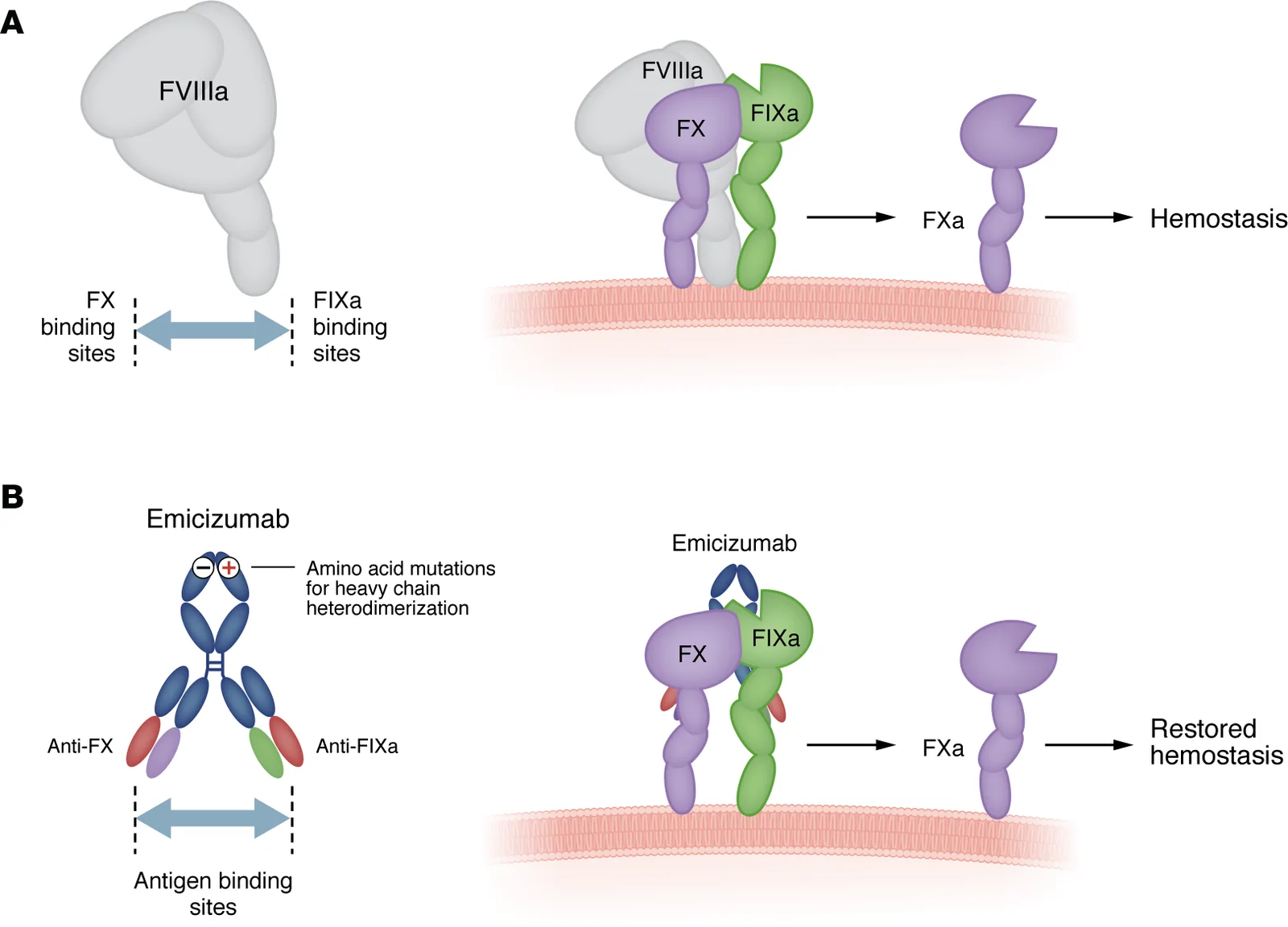
Emicizumab mimics the cofactor activity of FVIIIa by bridging FIXa and FX, thereby facilitating the generation of FXa. (**A**) FVIIIa is shown (gray), and the distance between the binding sites for FX and FIXa is depicted. The cofactor activity of FVIIIa requires positioning the catalytic site of FIXa (green) so that it will hydrolyze the cleavage site on FX (purple) to generate FXa. (**B**) Emicizumab is a bispecific IgG that binds the epidermal growth factor–like (EGF-like) domain 1 of FIX/FIXa with one arm and the EGF-like domain 2 of FX/FXa with the other arm. It was designed to have a common light chain (brown) and mutations in the Fc portion to facilitate heavy chain heterodimerization. These modifications reduced the number of possible permutations of antibodies during IgG assembly and enabled efficient production of the asymmetric bispecific IgG. Emicizumab mimics FVIIIa cofactor activity enabling the generation of FXa (9).

It was in this setting, in 2000, that Dr. Kunihiro Hattori, who led a group at Chugai Pharmaceuticals Co. Ltd. working on antibody-based therapies, conceived of a paradigm-shifting approach to hemophilia A therapy. Dr. Hattori noted that the distance between the two antigen-binding sites of human IgG is similar to that between the FIXa and FX-binding sites of FVIIIa. He reasoned that if an anti-FIXa/FX-bispecific antibody could be designed that places the catalytic center of FIXa at the FX cleavage site, such an antibody could potentially substitute for FVIIIa in catalyzing the activation of FX by FIXa (Figure 1). He and his team began work on a series of FIXa/FX-bispecific antibodies. In an effort led by Drs. Tomoyuki Igawa and Takehisa Kitazawa, the team immunized mice, rats, and rabbits with either FIXa or FX and cloned the genes of approximately 200 anti-FIXa antibodies and a similar number of anti-FX antibodies. They subsequently expressed these genes in HEK cells, creating a library of approximately 40,000 bispecific IgG combinations (9). These antibodies were screened for the ability to promote the generation of FXa following incubation with FIXa and FX. This program identified and subsequently characterized a lead bispecific IgG capable of promoting activation of FX to FXa (9).

In 2012, Dr. Kitazawa and colleagues published the description of a bispecific antibody capable of substituting for FVIIIa cofactor function (10). Although less potent than FVIIIa in promoting FXa generation, this bispecific antibody improved hemostasis in a nonhuman primate (NHP) model of acquired hemophilia A (10). The research team next optimized this bispecific by augmenting its binding properties to both FIXa and FX and improving its pharmacokinetics (11). They identified a common light chain compatible with the anti-FIXa and anti-FX heavy chains and incorporated additional improvements by protein engineering to optimize both manufacturing and functional properties (Figure 1). The active bispecific IgG had only a moderate affinity for binding FIX/FIXa and FX/FXa, allowing release of FXa from the antibody for participation in prothrombinase assembly (9). Molecular engineering of the bispecific antibody increased VIIIa-cofactor activity, enhanced solubility and decreased nonspecific binding to facilitate subcutaneous bioavailability, improved physicochemical stability to extend shelf life, and decreased immunogenicity score. The resultant antibody, termed ACE910 and later emicizumab, demonstrated activity in a thrombin generation assay comparable to that of FVIIIa, showed 86% bioavailability in NHPs with a half-life of approximately 3 weeks, and remained active in the presence of FVIII inhibitors (11). Subsequent studies of acquired hemophilia A in NHPs showed efficacy comparable to recombinant FVIIIa when used on demand in a model of acute bleeding (12) and when used as prophylaxis in a long-term model of joint bleeds (13). These preclinical studies provided robust proof of concept for the clinical development of ACE910 as a nonfactor alternative to FVIII replacement therapy in hemophilia A.

## Safe HAVENs: the clinical development of emicizumab

The emicizumab (ACE910) clinical development program focused initially on prophylactic administration for treatment of severe hemophilia A in patients with inhibitors, leveraging its longer half-life, subcutaneous administration, and FVIII inhibitor–resistant activity. Initial dose-escalation studies in healthy males published in 2016 showed a linear pharmacokinetic profile and a half-life of 4–5 weeks (14). In an interindividual dose-escalation study involving 18 patients with severe hemophilia A with or without FVIII inhibitors, the investigators found that weekly emicizumab shortened the activated partial thromboplastin time and reduced annualized bleeding rates equally in patients with or without FVIII inhibitors (15). No adverse events were detected.

The pivotal phase III trial that led to approval of emicizumab was HAVEN 1. It enrolled 109 people aged 12 years or older with hemophilia A with FVIII inhibitors and assessed of emicizumab’s efficacy, safety, and pharmacokinetics (16). Among participants who had been receiving episodic bypassing agents, emicizumab prophylaxis reduced the rate of treated bleeds by 87% compared with no prophylaxis (95% CI, 72.3–94.3; *P* < 0.0001). HAVEN 2 was a phase III trial that assessed pharmacokinetics, safety, and efficacy in 85 children younger than 12 years old with inhibitors. Participates received subcutaneous emicizumab 1.5 mg/kg weekly, 3 mg/kg every 2 weeks, and 6 mg/kg every 4 weeks. Annualized rates of bleeding were 0.3, 0.2, and 2.2, respectively, across these dosing groups (17). The trial showed that bleeding rates in this study were comparable to those reported from studies of recombinant FVIII in the absence of inhibitors. Moreover, target joints resolved in many participants, and some were able to have central venous access devices removed after transitioning from frequent intravenous infusions.

The success of emicizumab in patients with inhibitors prompted evaluation in patients without inhibitors. HAVEN 3 was a phase III, multicenter trial that evaluated subcutaneous emicizumab in individuals without FVIII inhibitors (18). Participants (*n* = 152) received emicizumab at 1.5 mg/kg once a week, 3 mg/kg once every 2 weeks, or no prophylaxis. The primary endpoint was the rate of treated bleeds. Compared with no prophylaxis, treated-bleed rates were 96% and 97% lower in the weekly and every-2-week emicizumab groups, respectively. More than half of participants receiving emicizumab had no treated bleeding events. In a nonrandomized intraindividual comparison, the annualized bleeding rate was 68% lower with emicizumab than with prior FVIII prophylaxis. No new FVIII inhibitors were reported during the study.

Additional clinical studies were performed to further expand the therapeutic scope of emicizumab. HAVEN 4 demonstrated the efficacy, safety, and pharmacokinetics of emicizumab given every 4 weeks in patients with severe congenital hemophilia A or hemophilia A with inhibitors (19). HAVEN 5 evaluated patients across the Asia-Pacific region (20). HAVEN 6 tested emicizumab in patients with nonsevere hemophilia A (21), and HAVEN 7 showed that emicizumab decreased bleeding in infants with severe hemophilia A without inhibitors (22).

## Perspective

Emicizumab has been a disruptive force in hemophilia A treatment. It is currently by far the most widely used agent for prophylactic treatment of hemophilia A, outpacing its closest competitor by nearly an order of magnitude. Since its launch in 2017 (marketed as Hemlibra) it has not only become a first-line therapy, but has consolidated a historically fragmented hemophilia A market around a single bispecific antibody treatment. It has increased the percentage of patients with moderate and even mild hemophilia A who receive continuous prophylaxis and has extended prophylaxis into resource-limited settings. There is evidence that emicizumab has contributed to decreased inhibitor development in the hemophilia A population (23). And, most importantly, it has dramatically decreased bleeding, reduced hospitalizations, and improved the quality of life for tens of thousands of individuals.

## Conflict of interest

The author has declared that no conflict of interest exists.

## Version 1 09/09/2026

Electronic publication

## Version 2 09/15/2026

Electronic publication
